# Prognostic impact of atrial fibrillation in patients undergoing transcatheter aortic valve implantation

**DOI:** 10.1016/j.hroo.2024.12.016

**Published:** 2025-01-10

**Authors:** Jakob J. Reichl, Thorald Stolte, Jasper Boeddinghaus, Max Wagener, Gregor Leibundgut, Patrick Badertscher, Christian Sticherling, Michael Kühne, Christoph Kaiser, Felix Mahfoud, Thomas Nestelberger

**Affiliations:** Department of Cardiology and Cardiovascular Research Institute Basel (CRIB), University Hospital Basel, University of Basel, Basel, Switzerland

**Keywords:** Anticoagulation, Arrhythmia, Atrial fibrillation, Stroke, TAVI

## Abstract

**Background:**

Atrial fibrillation (AF) is the most common arrhythmia and an important risk factor for adverse cardiac outcomes, including heart failure and stroke. Moreover, AF has been linked to worse outcomes after transcatheter aortic valve implantation (TAVI). Real-world data on the impact of AF on outcomes after TAVI remain limited.

**Objective:**

To assess the impact of AF on periprocedural and short-term outcomes after TAVI.

**Methods:**

Patients undergoing TAVI at a tertiary center were consecutively included in a prospective registry. Cardiac rhythm at baseline was assessed using 12-lead ECGs. The primary outcome was all-cause mortality at 30 days. Secondary outcomes included all-cause mortality at 1 year, stroke at 30 days and 1 year, and procedural success, defined as freedom from periprocedural mortality, surgical re-interventions, re-interventions of the aortic valve, major access site complications, and periprocedural bleedings until discharge.

**Results:**

Among 1655 patients undergoing TAVI, 428 patients (25.6%) had preexisting AF, and 77 patients (4.6%) were diagnosed with new-onset AF during hospitalization for TAVI. AF was not associated with higher mortality at 30 days (3.7% vs 2.0%; *P* = .054, adjusted hazard ratio [aHR], 1.8 [95% confidence interval (CI), 0.9–3.4]), but at 1 year (13.8% vs 8.4%; *P* = .001; aHR, 1.6 [95%CI, 1.2–2.2]). The stroke rate was higher in patients with AF at 30 days (5.9% vs 2.7%; *P* = .003; aHR, 2.1 [95%CI, 1.2–3.5]) and at 1 year (7.1% vs 3.8%; *P* = .005; aHR, 1.8 [95%CI, 1.2–2.9]). At discharge, 452 patients (89.5%) with AF received oral anticoagulation. After adjusting for anticoagulant therapy, the difference in stroke risk at 30 days (5.7% vs 2.3%; *P* = .058) and 1 year (6.8% vs 4.2%; *P* = .165) was no longer significant. Patients with AF experienced more major or life-threatening bleeding complications (14.2% vs 10.6%; *P* = .043). There were no differences in procedural success between patients with and those without AF (78.8% vs 78.3%; *P* = .886).

**Conclusion:**

AF was associated with increased mortality at 1 year and higher rates of stroke and major bleeding at 30 days and 1 year after TAVI.

**Key findings**

▪ Atrial fibrillation (AF) is a common arrhythmia in patients undergoing TAVI.
▪ AF is associated with increased mortality, stroke, and major bleeding.
▪ Sufficient anticoagulation before TAVI decreases stroke risk.

## Introduction

Aortic stenosis (AS) is among the most prevalent valve pathologic condition in patients aged 75 years and older, affecting up to 20% of this population.^1^ Untreated patients with severe aortic stenosis face high rates of mortality and morbidity.^2^ Transcatheter aortic valve implantation (TAVI) has emerged as a safe and effective treatment for severe aortic valve stenosis, particularly in elderly or high-risk surgical patients, and is now recommended as the first-line treatment for these groups by international guidelines.^3^, ^4^, ^5^

Atrial fibrillation (AF) is the most common cardiac arrhythmia in the elderly and is associated with mortality and morbidity.^6^ AF significantly contributes to adverse cardiac outcomes such as heart failure and stroke.^7^ AF has also been associated with adverse outcomes after TAVI, particularly if newly detected after the procedure.^8^ Previous studies report the prevalence of preexisting AF in TAVI patients ranging from 16% to 51.1%.^9^ Additionally, new-onset AF is frequently found after TAVI, with a recent meta-analysis by Ryan et al^10^ reporting a postprocedural incidence of 9.9%. Given the increasing number of patients undergoing TAVI, it is crucial to further evaluate the impact of AF on post-TAVI outcomes. Whether AF directly contributes to adverse outcomes or whether patients with AF have more comorbidities, representing a population at inherently greater risk, remains unclear. Therefore, this study aimed to assess the impact of both preexisting and new-onset AF on outcomes in patients undergoing TAVI.

## Methods

### Study design and patient cohort

Patients who underwent TAVI at the University Hospital Basel, Switzerland, were included in a prospective national database, as part of the Swiss TAVI registry, mandated by the Swiss health authorities (NCT01368250). The Swiss TAVI registry is a multicentric study and has been described in detail previously. It has been approved by the local cantonal ethics committee and the institutional review boards of all participating sites. All patients provided written informed consent for study participation and prospective follow-up assessment. The research reported in this article adhered to the Declaration of Helsinki.

### Data collection and clinical endpoints

Patient-related data, such as baseline characteristics, peri-procedural information, and follow-up records, were prospectively collected and recorded in a web-based database. Clinical follow-up data were obtained through standardized interviews, documentation from referring physicians, and hospital discharge summaries after 30 days and yearly thereafter. All adverse events were systematically collected and adjudicated by a dedicated clinical event committee. Patients included in this study were categorized based on cardiac rhythm at baseline, a history of AF, as well as cardiac rhythm at discharge. Patients were excluded if preprocedural cardiac rhythm had not been recorded. All patients underwent systematic 12-channel electrocardiogram (ECG) recordings during pre-interventional diagnostic workup, on admission, and daily until discharge, as well as continuous telemetry monitoring post-transcatheter aortic valve implantation (72 hours for patients with new-onset left bundle branch block and 48 hours for all remaining patients). Long-term ambulatory ECG monitoring (eg, Holter monitoring or loop recorders) was performed if clinically indicated. New-onset AF was defined as AF over 30 seconds during patient monitoring, AF in a 12-lead ECG, or Holter-ECG in patients without a history of prior AF.

For patients with preexisting AF on non-vitamin K antagonist oral anticoagulants (NOACs), anticoagulation was paused 48 hours before TAVI and restarted 24 hours after the procedure if no significant bleeding occurred. For those on vitamin K antagonists, the International Normalized Ratio was adjusted to <2.0 at the time of intervention. Bridging with low-molecular-weight heparin was reserved for patients with mechanical valve replacements or high thromboembolic risk. Six hours after intervention heparin or low-molecular-weight heparin in a prophylactic dose was administered. Anticoagulation, either with NOAK or with Vitamin K antagonists, was restarted the morning after the procedure.

## Outcomes

Outcomes of interest were defined as described below according to Valve Academic Research Consortium (VARC) III guidelines.^11^ The main outcome was all-cause mortality at 30 days. Secondary outcomes included all-cause mortality at 1 year, stroke rates at 30 days and 1 year, as well as procedural success, a combined endpoint including freedom of periprocedural mortality, which we defined as death during the intervention, freedom of valve embolization, freedom of surgical or interventional re-interventions of the aortic valve, or major access-related complications. Additional outcomes were periprocedural acute kidney injury, periprocedural bleeding, including minor and major bleedings regardless of bleeding location until discharge, permanent pacemaker implantation, length of in-hospital stay and in intensive care units, as well as major or life-threatening bleedings, according to Valve Academic Research Consortium III criteria, after 30 days.

### Statistical analysis

Categorical variables are presented as frequencies and percentages. Continuous variables are represented as mean values ± standard deviation if normal distribution is apparent. In non-normally distributed continuous variables, median values are presented as the 25^th^ and 75^th^ percentile. Normal distribution was tested using the Kolmogorov-Smirnov test. Statistical comparisons for normally distributed continuous variables were conducted using Fisher's paired *t*-test, and non-normally distributed variables were analyzed using the Mann-Whitney *U*-test. Differences in ordinal scaled data were assessed using Pearson’s χ^2^ test. Survival analysis was carried out using the Kaplan-Meier method, and differences in survival were evaluated using the log-rank Cox regression. *P* < .05 was considered statistically significant. To account for possible confounders, an adjusted hazard ratio (aHR) was calculated, using a linear regression model consisting of age, sex, AF, coronary artery disease, prior myocardial infarction or cardiac surgery, peripheral artery disease, chronic kidney disease, and prior implantation of a permanent cardiac pacemaker before TAVI. All statistical analyses were performed using R 4.2.3 (R Foundation for Statistical Computing, Vienna, Austria).

## Results

### Patient characteristics

Among 1655 patients undergoing TAVI from September 2011 to April 2024, preexisting AF was documented in 428 (25.6%) patients, whereas 77 patients (4.6%) developed new-onset AF during their hospitalization. Baseline characteristics are represented in Table 1.

**Table 1:** Baseline characteristics

	Overall (n = 1655)	Atrial fibrillation (n = 505)	No atrial fibrillation (n = 1154)	*P*
**Sex**				.002
**Female**	782 (48.3%)	213 (42.5%)	579 (50.8%)	
**Age, y**	82.2 (±6.2)	83.1 (5.8)	81.8 (6.3)	**<.001**
**BMI**	26.7 (23.3, 29.2)	26.4 [23.4, 29.5]	26.0 [23.3, 29.2]	.190
**EuroScore II**	3.7 [1.3, 4.4]	2.8 [1.6, 5.5]	2.1 [1.2, 4.1]	**<.001**
**STS score**	4.8 [2.2, 5.6]	3.8 [2.6, 5.9]	3.2 [2.1, 5.4]	**<.001**
**Hypertension**	1323 (80.6%)	405 (80.8%)	918 (80.5%)	.937
**Diabetes**	473 (28.8%)	143 (28.5%)	330 (28.9%)	.914
**Dyslipidemia**	981 (59.8%)	273 (54.5%)	708 (62.1%)	**.004**
**Coronary artery disease (CAD)**	908 (55.3%)	282 (56.3%)	626 (54.9%)	.644
**Prior myocardial infarction (MI)**	277 (16.9%)	95 (19.0%)	182 (16.0%)	.155
**Prior percutaneous coronary intervention (PCI)**	568 (34.6%)	174 (34.7%)	394 (34.6%)	.992
**Prior cardiac surgery**	162 (9.9%)	57 (11.4%)	105 (9.2%)	.206
**Permanent cardiac pacemaker**	170 (10.4%)	69 (13.8%)	101 (8.9%)	**.004**
**Permanent intracardiac defibrillator**	9 (0.5%)	6 (1.2%)	3 (0.3%)	.046
**Cerebrovascular disease**	202 (12.3%)	71 (14.2%)	131 (11.5%)	.150
**Peripheral artery disease (PAD)**	275 (16.8%)	86 (17.2%)	189 (16.6%)	.825
**Chronic kidney disease (CKD)**	1053 (64.5%)	349 (70.1%)	704 (62.0%)	**.002**
**Dialysis**	37 (2.3%)	10 (2.0%)	27 (2.4%)	.774
**COPD**	157 (9.6%)	49 (9.8%)	108 (9.5%)	.918

BMI = body mass index; COPD = chronic obstructive pulmonary disease; STS-Score = Society of Thoracic Surgeons—Mortality Score.

Patients with AF were older (83.1 vs 81.8 years; *P* < .001), more often male (57.5% vs 49.2%; *P* = .012), and had higher periprocedural risk scores, including an elevated EuroScore II (2.8 vs 2.1 points; *P* < .001) and Society of Thoracic Surgeons Mortality Score (3.8 vs 3.2 points; *P* < .001). There were no differences in the prevalence of common cardiovascular comorbidities such as coronary artery disease, hypertension, diabetes, or cerebrovascular disease. However, patients with AF had higher rates of chronic kidney disease, defined as an estimated glomerular filtration rate < 60 mL/min (70.1% vs 62.0%; *P* = .002) at baseline, and more frequent prior permanent pacemaker implantation (16.3% vs 8.3%; *P* = .004).

### Imaging findings

Echocardiographic findings are displayed in Table 2. Patients with AF had lower left ventricular ejection fraction at baseline (55% vs 59%; *P* < .001) and lower mean aortic valve gradients (41 mm Hg vs 45 mm Hg, *P* < .001). Additionally, AF patients were more likely to exhibit signs of pulmonary hypertension, as indicated by a systolic pulmonary artery pressure of >36 mm Hg (17.5% vs 10.5%; *P* < .001) and right ventricular dysfunction (23.6% vs 8.6%; *P* < .001) at baseline. Larger valve sizes were used in AF patients (27 mm vs 26 mm; *P* < .001), although there were no differences in device type or vascular access.

**Table 2:** Echocardiographic and interventional findings

	Overall (n = 1655)	Atrial fibrillation (n = 505)	No atrial fibrillation (n = 1154)	*P*
**LVEF at baseline, %**	58.0 [45.0, 62.0]	55.0 [45.0, 60.0]	59.0 [47.0, 63.0]	**.001**
**Aortic valve area (AVA)**	44.0 [36.0, 52.0]	41.0 [31.0, 50.2]	45.0 [38.0, 53.0]	**.001**
**Mean aortic valve gradient, mm Hg**	66.0 [48.0, 80.0]	59.5 [42.0, 77.0]	68.0 [51.0, 80.0]	**<.001**
**Maximum aortic valve gradient, mm Hg**	0.8 [0.6, 0.9]	0.7 [0.6, 0.9]	0.8 [0.6, 0.9]	**<.001**
**Left ventricular mass, g**	189 (13.2%)	104 (23.6%)	85 (8.6%)	.970
**Left ventricular mass-index, g/m**^**2**^	179 (12.5%)	77 (17.5%)	102 (10.3%)	.594
**Left ventricular end-diastolic diameter, mm**	193.0 [156.0, 235.0]	194.0 [167.5, 231.5]	193.0 [156.0, 235.0]	.226
**Left ventricular end-systolic diameter, mm**	108.0 [91.0, 128.0]	109.0 [91.0, 128.0]	108.0 [91.0, 128.0]	.577
**Right ventricular dysfunction at baseline**	46.0 [41.0, 51.0]	46.0 [42.0, 52.0]	45.7 [40.5, 50.0]	**<.001**
**Pulmonary hypertension at baseline**^a^	31.0 [26.0, 37.0]	32.0 [26.8, 38.0]	30.0 [26.0, 37.0]	**<.001**
**Valve size**	26.0 [25.0, 29.0]	27.0 [25.0, 29.0]	26.0 [25.0, 29.0]	**<.001**
**Device type**				.141
Self-expandable	1167 (70.5%)	348 (68.9%)	819 (70.9%)	
Balloon-expandable	152 (30.1%)	115 (27.2%)	317 (27.5%)	
**Vascular access**				.970
Transfemoral access	1434 (86.6%)	437 (87.2%)	997 (86.4)	
Transapical access	163 (9.8%)	50 (9.9%)	113 (9.8%)	
Subclavian access	39 (2.3%)	12 (2.4%)	27 (2.3%)	
Direct aortic access	5 (0.3%)	2 (3.9%)	3 (0.25%)	

LVEF = left ventricular ejection fraction.

a Defined as a systolic pulmonary artery pressure of ≥36 mm Hg in transthoracic echocardiography.

### Medication

Patients with AF were more often treated with oral anticoagulants (vitamin K-antagonists or NOAC) compared with those without AF (83.5% vs 13.4%; *P* < .001). They were less likely to be on low-dose aspirin (21.4% vs 65.2%; *P* < .001) or a P2Y12-inhibitor (13.2% vs 18.5%; *P* = .010) at baseline. At discharge, patients with AF were more frequently on oral anticoagulation (89.5% vs 19.2%; *P* < .001), but less likely to be taking aspirin (20.2% vs 80.5%; *P* < .001) or P2Y12-inhibitors (36.2% vs 61.4%; *P* < .001). Table 3 summarizes findings of medications at baseline and discharge.

**Table 3:** Medication at baseline and discharge

	Atrial fibrillation (n = 505)	No atrial fibrillation (n = 1154)	*P*
**Low-dose aspirin**			
Baseline	21.4%	65.2%	<.001
Discharge	20.2%	80.5%	<.001
**P2Y12-Inhibitors**			
Baseline	13.2%	18.5%	.010
Discharge	36.2%	61.4%	<.001
**Oral anticoagulation**^a^			
Baseline	83.5%	13.4%	<.001
Discharge	89.5%	19.2%	<.001

a Oral anticoagulation = Vitamin K-antagonists or NOAC.

### Outcomes after TAVI

**Figure 1:**
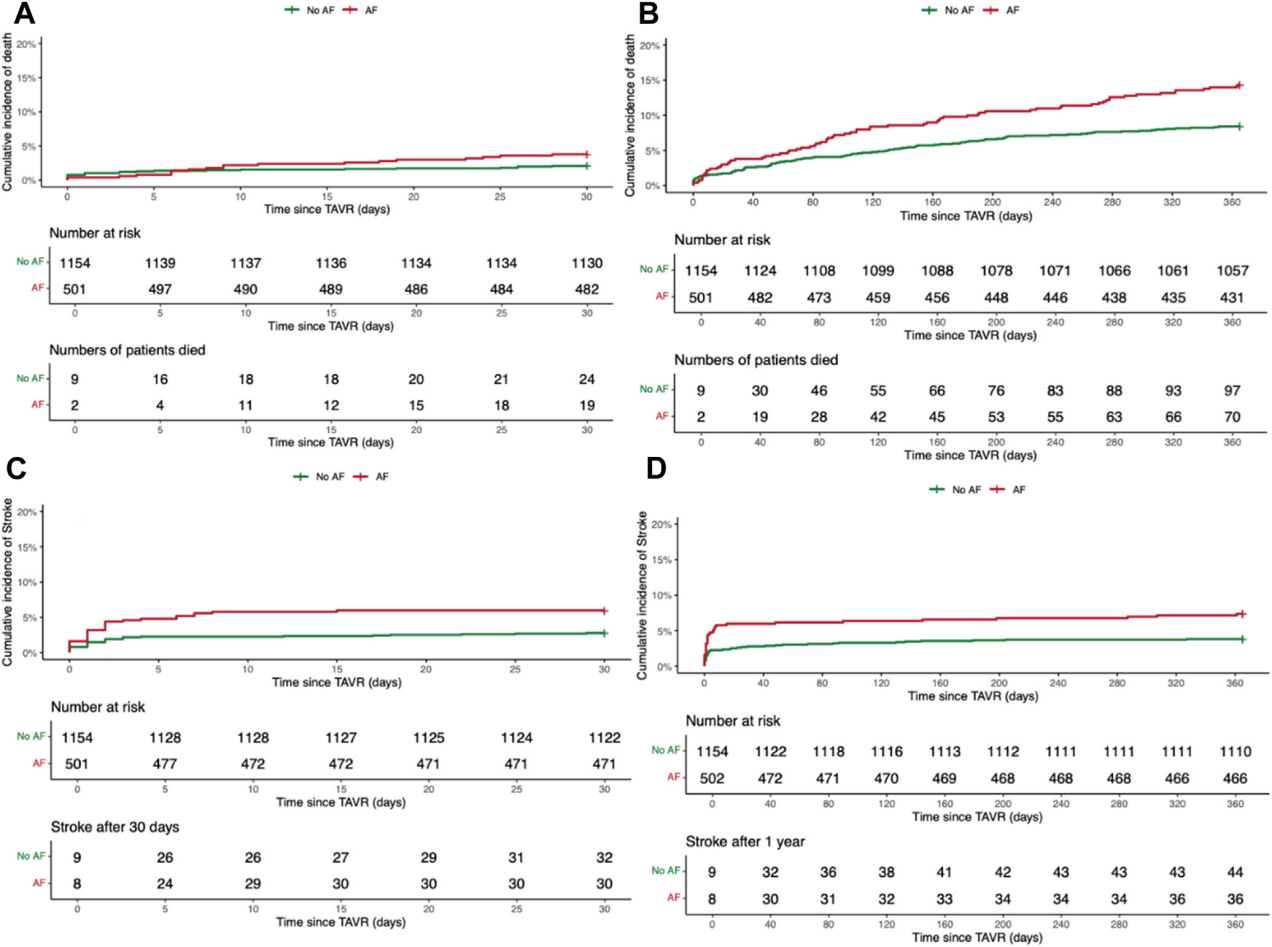
**A–D:** Kaplan-Meier-Curves showing mortality after 30 days (**A**), 1 year (**B**), stroke after 30 days (**C**), and 1 year (**D**).

All-cause mortality was significantly higher for patients with AF at 30 days (3.7% vs 2.0%; hazard ratio, 1.8 [95%CI, 1.0–3.3]; *P* = .046; aHR, 1.8 [95%CI, 0.9–3.3]; *P* = .054), and at 1 year (13.8% vs 8.4%; HR, 1.7 [95%CI, 1.2–2.3]; *P* < .001; aHR, 1.6 [95%CI, 1.2–2.2]; *P* = .001). After adjusting for confounders, the difference in 30-day mortality rates was no longer significant (aHR, 1.8 [95%CI, 0.9–3.3]; *P* = .054). Stroke risk was higher for AF patients at 30 days (5.9% vs 2.7%; HR, 2.1 [95%CI, 1.3–3.6]; *P* = .001; aHR, 2.1 [95%CI, 1.3–3.5]; *P* = .003), and at 1 year (7.1% vs 3.8%; HR 1.9 [95%CI, 1.2–3.0]; *P* = .001; aHR, 1.8 [95%CI, 1.2–2.9); *P* = .005). Among patients on oral anticoagulation at baseline, no difference in stroke rates was observed at 30 days (5.7% vs 2.3%; *P* = .058) or at 1 year (6.8% vs 4.2%; *P* = .165). We observed no difference in the combined endpoint of periprocedural success (78.8% vs 78.3%; *P* = .886), which was documented in 395 patients with AF and 944 patients without AF. No difference was found in the incidence of vascular complications (12.7% vs 10.7%; *P* = .304), periprocedural mortality (0.4% vs 0.7%; *P* = .584), valve embolization (0.2% vs 2.2%; *P* = .884), or surgical interventions (0.2% vs 0.5%; *P* = .609). The incidence of periprocedural acute kidney injury was similar between both groups (1.8% vs 1.4%; *P* = .809), and no significant difference was found in minor and major bleeding complications regardless of location (16.4% vs 14.4%; *P* = .333) until discharge. However, at 30 days, patients with AF experienced a higher incidence of life-threatening or major bleedings compared with those without AF (14.2% vs 10.6%; *P* = .043). After excluding patients with prior pacemaker implantation, no difference was seen in postprocedural permanent pacemaker implantation at discharge between patients with and without AF (14.6% vs 17.9%; *P* = .780). In-hospital stay was similar between groups (8.2 vs 8.7 days; *P* = .38). Procedural outcomes are shown in Table 4, and stroke and mortality outcomes are summarized in Table 5. Kaplan-Meier curves illustrating mortality and stroke outcomes are presented in Figure 1.

**Table 4:** Periprocedural outcomes after TAVI

	Atrial fibrillation (n = 505)	No atrial fibrillation (n = 1154)	*P*
**Periprocedural success**	395 (78.8%)	904 (78.3%)	.886
**Periprocedural mortality**	2 (0.4%)	9 (0.7%)	.584
**Valve embolization**	1 (0.2%)	26 (2.2%)	.884
**Surgical intervention**	1 (%)	6 (0.51%)	.609
**Vascular complication**	64 (12.7%)	124 (10.7%)	.304
**Periprocedural bleeding**	83 (16.4%)	166 (14.4%)	.333
**Periprocedural acute kidney injury**	9 (1.8%)	17 (1.4%)	.809

**Table 5:** Mortality rates and stroke after TAVI

	Atrial fibrillation (n = 505)	No AF (n = 1154)	Hazard ratio (95% CI)	Unadjusted *P*	Adjusted hazard ratio (aHR) (95% CI)	Adjusted *P*
**Mortality (30 days)**	3.7%	2.0%	1.8 (1.0–3.3)	.049	1.8 (0.9–3.3)	.054
**Mortality (1 year)**	13.8%	8.4%	1.7 (1.2–2.3)	<.001	1.6 (1.2–2.2)	.001
**Stroke (30 days)**	6.1%	2.9%	2.1 (1.3–3.6)	.001	2.1 (1.2–3.5)	.003
**Stroke (1 year)**	7.1%	3.8%	1.9 (1.2–3.0)	.001	1.8 (1.2–2.9)	.005

Outcomes were adjusted for age, sex, coronary artery disease, prior myocardial infarction, peripheral artery disease, prior cardiac surgery of any kind, as well as chronic kidney disease and permanent pacemaker.

## Discussion

This study aimed to assess the impact of AF on outcomes after TAVI. Our findings contribute to the growing body of evidence on long-term outcomes after TAVI. We report the following main findings:

First, AF is prevalent in approximately one fourth of patients undergoing TAVI and was newly diagnosed in 4.6% of patients. Second, patients with AF were generally older, more often male, and had more cardiovascular comorbidities. Third, procedural outcomes including length of stay in hospital were similar; patients with AF experienced a higher incidence of major bleeding complications after 30 days. Fourth, AF was associated with higher all-cause mortality after 1 year, as well as higher stroke risk after 1 year and 30 days, even after adjusting for common confounders. Notably, oral anticoagulation was prescribed to most AF patients and lowered the risk of stroke compared with patients not receiving oral anticoagulation.

Our findings extend and corroborate prior findings from other cohorts. Prior studies have shown higher mortality rates in patients with preexisting AF attributable to multiple factors, such as hemodynamic and functional impairment, bleeding events, as well as thromboembolic events.^8^^,^^12^^,^^13^ In our study, patients with preexisting AF had a lower median left ventricular ejection fraction, suggesting worse cardiovascular status, possibly contributing to their poorer outcomes. Additionally, we found higher rates of major bleeding events at 30 days among patients with AF, contributing to a higher risk of mortality, a finding that has been reported in earlier studies.^14^

Cerebrovascular complications, such as stroke, are a major complication of TAVI and contribute to postprocedural mortality, accounting for up to 11% of deaths after TAVI.^15^ In our study, AF was associated with an overall higher stroke risk at 30 days and 1 year. Our results echo a recent meta-analysis conducted by Nso et al,^16^ which found higher stroke risk only in patients with new-onset AF, not in those with preexisting AF. We observed that the stroke risk decreased significantly when oral anticoagulation was prescribed, resulting in lower and more comparable stroke rates between the 2 groups. Approximately 83.5% of patients with AF were on oral anticoagulants, and 89.5% of patients with preexisting AF received an anticoagulant at discharge. The gap between these 2 figures raises the concern of undertreatment before TAVI. Undertreatment of AF in the elderly has been recognized as a major concern, with rates of undertreatment reported between 10.0% and 45.1%.^17^ This is particularly concerning given the substantial benefit that adequate anticoagulant therapy provides demonstrated in a recent meta-analysis.^18^

Although acute bleeding rates until discharge did not differ between groups, AF was associated with a higher risk of early major and life-threatening bleeding complications after 30 days. Although advances TAVI techniques and technologies, such as smaller sheath sizes and the inclusion of patients with lower periprocedural risk, have reduced bleeding complications, these remain a matter of concern. This is consistent with findings from the PARTNER trials 1–3 (Placement of AoRTic TraNscathetER Valve Trial^4^^,^^19^^,^^20^) that bleeding complications continue to be a major concern. Efforts to identify patients at high risk for major bleeding complications have been made,^21^^,^^22^ but up until now, these tools have not resulted in higher detection rates of patients at risk. Further research is needed to develop effective risk stratification tools and to optimize periprocedural antithrombotic strategies for high-risk patients.

## Limitations

Our study has several limitations. As a retrospective analysis of prospectively enrolled patients, there may be limitations in statistical power, potentially leading to underestimation of differences in some outcomes. Additionally, because the data were drawn from a single-center database, the generalizability of our results should be interpreted with caution, and external validation is warranted. Despite these limitations, our study provides valuable real-world data collected over more than a decade. Finally, despite using standard care of practice for AF screening in patients undergoing TAVI, long-term monitoring was only performed if clinically indicated, which may have resulted in an underestimation of the true prevalence of newly diagnosed AF.

## Conclusion

In conclusion, AF is associated with increased all-cause mortality, stroke, and bleeding complications in patients undergoing TAVI. Appropriate anticoagulation therapy may impact the risk of periprocedural stroke in patients with AF.

## Disclosures

Thomas Nestelberger has received research support from the Swiss National Science Foundation (P400PM_191037/1), the Prof. Dr. Max Cloëtta Foundation, the Margarete und Walter Lichtenstein-Stiftung (3MS1038), and the University Hospital Basel as well as speaker honoraria/consulting honoraria from Edwards Lifesciences, Siemens, Beckman Coulter, Bayer, Ortho Clinical Diagnostics and Orion Pharma, outside the submitted work. Jasper Boeddinghaus is supported by an Edinburgh Doctoral College Scholarship and research grants from the University of Basel, the University Hospital of Basel, the Division of Internal Medicine, the Swiss Academy of Medical Sciences, the Gottfried and Julia Bangerter-Rhyner Foundation, the Swiss National Science Foundation, and the “Freie Akademische Gesellschaft” and has received honoraria from Siemens, Roche Diagnostics, Ortho Clinical Diagnostics, Quidel Corporation, and Beckman Coulter, and travel support from Medtronic and Cordis, all outside the submitted work. Felix Mahfoud is supported by Deutsche Gesellschaft für Kardiologie (DGK), Deutsche Forschungsgemeinschaft (SFB TRR219, Project-ID 322900939), and Deutsche Herzstiftung. Saarland University has received scientific support from Ablative Solutions, Medtronic and ReCor Medical. Until May 2024, Felix Mahfoud has received speaker honoraria/consulting fees from Ablative Solutions, Amgen, Astra-Zeneca, Bayer, Boehringer Ingelheim, Inari, Medtronic, Merck, ReCor Medical, Servier, and Terumo. All other authors declare that they have no conflict of interest with this study.

## References

[1] Osnabrugge R.L.J., Mylotte D., Head S.J. (2013). Aortic stenosis in the elderly. J Am Coll Cardiol.

[2] Généreux P., Sharma R.P., Cubeddu R.J. (2023). The mortality burden of untreated aortic stenosis. J Am Coll Cardiol.

[3] Vahanian A., Beyersdorf F., Praz F. (Ed 2022). 2021 ESC/EACTS Guidelines for the management of valvular heart disease: developed by the Task Force for the management of valvular heart disease of the European Society of Cardiology (ESC) and the European Association for Cardio-Thoracic Surgery (EACTS). Rev Esp Cardiol Engl.

[4] Leon M.B., Smith C.R., Mack M. (2010). Transcatheter aortic-valve implantation for aortic stenosis in patients who cannot undergo surgery. N Engl J Med.

[5] Gilard M., Eitchaninoff H., Iung B. (2012). Registry of transcatheter aortic-valve implantation in high-risk patients. N Engl J Med.

[6] Mairesse G.H., Moran P., Van Gelder I.C. (2017). Screening for atrial fibrillation: a European Heart Rhythm Association (EHRA) consensus document endorsed by the Heart Rhythm Society (HRS), Asia Pacific Heart Rhythm Society (APHRS), and Sociedad Latinoamericana de Estimulación Cardíaca y Electrofisiología (SOLAECE). EP Eur.

[7] Ball J., Carrington M.J., McMurray J.J.V., Stewart S. (2013). Atrial fibrillation: profile and burden of an evolving epidemic in the 21st century. Int J Cardiol.

[8] Biviano A.B., Nazif T., Dizon J. (2016). Atrial fibrillation is associated with increased mortality in patients undergoing transcatheter aortic valve replacement: insights from the PARTNER Trial. Circ Cardiovasc Interv.

[9] Tarantini G., Mojoli M., Urena M., Vahanian A. (2017). Atrial fibrillation in patients undergoing transcatheter aortic valve implantation: epidemiology, timing, predictors, and outcome. Eur Heart J.

[10] Ryan T., Grindal A., Jinah R. (2022). New-onset atrial fibrillation after transcatheter aortic valve replacement. JACC Cardiovasc Interv.

[11] Généreux P., Piazza N., Alu M.C. (2021). Valve academic research consortium 3: updated endpoint definitions for aortic valve clinical research. J Am Coll Cardiol.

[12] Chopard R., Teiger E., Meneveau N. (2015). Baseline characteristics and prognostic implications of pre-existing and new-onset atrial fibrillation after transcatheter aortic valve implantation: results from the FRANCE-2 registry. JACC Cardiovasc Interv.

[13] Tarantini G., Mojoli M., Windecker S. (2016). Prevalence and impact of atrial fibrillation in patients with severe aortic stenosis undergoing transcatheter aortic valve replacement: an analysis from the SOURCE XT Prospective Multicenter registry. JACC Cardiovasc Interv.

[14] Arrotti S., Sgura F.A., Leo G. (2024). Atrial fibrillation before and after transcatheter aortic valve implantation: short- and long-term clinical implications. J Cardiovasc Med (Hagerstown).

[15] Moreno R., Calvo L., Salinas P. (2011). Causes of peri-operative mortality after transcatheter aortic valve implantation: a pooled analysis of 12 studies and 1223 patients. J Invasive Cardiol.

[16] Nso N., Emmanuel K., Nassar M. (2022). Impact of new-onset versus pre-existing atrial fibrillation on outcomes after transcatheter aortic valve replacement/implantation. Int J Cardiol Heart Vasc.

[17] Sussman M., Barnes G.D., Guo J.D. (2022). The burden of undertreatment and non-treatment among patients with non-valvular atrial fibrillation and elevated stroke risk: a systematic review. Curr Med Res Opin.

[18] Patti G., Lucerna M., Pecen L. (2017). Thromboembolic risk, bleeding outcomes and effect of different antithrombotic strategies in very elderly patients with atrial fibrillation: a sub-analysis from the PREFER in AF (PREvention oF Thromboembolic Events-European Registry in Atrial Fibrillation). J Am Heart Assoc.

[19] Mack M.J., Leon M.B., Thourani V.H., Makkar R., Kodali S.K., Russo M. (2019). Transcatheter aortic-valve replacement with a balloon-expandable valve in low-risk patients. N Engl J Med.

[20] Leon M.B., Smith C.R., Mack M.J. (2016). Transcatheter or surgical aortic-valve replacement in intermediate-risk patients. N Engl J Med.

[21] Garot P., Neylon A., Morice M.C. (2022). Bleeding risk differences after TAVI according to the ARC-HBR criteria: insights from SCOPE 2. EuroIntervention.

[22] Albabtain M.A., Arafat A.A., Alghasoon H., Abdelsalam W., Almoghairi A., Alotaiby M. HAS-BLED score for prediction of bleeding and mortality after transcatheter aortic valve replacement. Braz J Cardiovasc Surg. https://cdn.publisher.gn1.link/bjcvsorg/pdf/0102-7638-rbccv-38-01-0037.pdf.

